# Human *Rickettsia felis* Infection, Canary Islands, Spain

**DOI:** 10.3201/eid1112.050711

**Published:** 2005-12

**Authors:** Jose-Luis Pérez-Arellano, Florence Fenollar, Alfonso Angel-Moreno, Margarita Bolaños, Michele Hernández, Evora Santana, Marion Hemmersbach-Miller, Antonio-M Martín, Didier Raoult

**Affiliations:** * Hospital Universitario Insular de Las Palmas, Canary Islands, Spain; † Universidad de Las Palmas de Gran Canaria, Canary Islands, Spain; ‡ Université de la Mediterranée, Marseille, France

**Keywords:** Rickettsia felis, human infection, fleas, Western-Blot serology, Canary Island, Spain, dispatch

## Abstract

We report the first cases of human infection by *Rickettsia felis* in the Canary Islands. Antibodies against *R. felis* were found in 5 adsorbed serum samples from 44 patients with clinically suspected rickettsiosis by Western blot serology. Fleas from 1 patient's dog were positive for *R. felis* by polymerase chain reaction.

*Rickettsia felis* is an intracellular bacterium (genus *Rickettsia*, spotted fever group [SFG]) (*1**,**2*). Its biological cycle involves the cat flea (*Ctenocephalides felis*) as the main vector (*3*). *R. felis* has been found in *C. felis* and *C. canis* in the Americas, Europe, Africa, Asia, and Oceania (*1**,**3**–**6*). Human disease caused by *R. felis* was unknown until 1994 (*4*). Since then, *R. felis* infection has been reported in Mexico (3 patients) (*7*), Germany (1 patient) (*8*), Brazil (2 patients) (*1*), and France (2 patients) (*1*). The clinical manifestations of the disease include high fever, rash, and elevation of liver enzymes (*1**,**4**,**7*). Exposure to fleas or to flea-prone animals is sometimes recorded (*7**,**8*).

On the Canary Islands (Atlantic islands of Spain), autochthonous cases of murine typhus have been reported (*9*). Although we suspected that some patients with a clinical picture of murine typhus actually had *R. felis* infection, we were not able to confirm this hypothesis. Therefore, 44 serum samples from 44 patients from the Canary Islands with suspected murine typhus were sent to the Unité des Rickettsies in Marseille, France, for specific serologic tests. Here, we describe the first 5 human infections caused by *R. felis* on the Canary Islands.

## The Study

Forty-four patients were recruited for a prospective study of fever of intermediate duration (i.e., fever without focal symptoms lasting 7–28 days). Demographic, clinical, and laboratory data were collected for all patients. Chest radiographs and blood and urine cultures were taken. Antibodies against *R. typhi* were tested by direct immunofluorescence test (bioMérieux, Marcy L'Etoile, France) in the Canary Islands. Among the 44 patients, 24 showed a positive serologic result. Antibodies against other agents (*Coxiella burnetii*, *R. conorii*, *Leptospira* spp., Epstein-Barr virus, cytomegalovirus, HIV, and hepatitis B virus) were also tested; all were negative.

To search for evidence of infection with *R. felis*, all serologic results were confirmed by microimmunofluorescence (MIF) in France, as previously described (*10*). Systematic testing of SFG rickettsia antigens present in Europe and Africa was performed in parallel. The MIF procedure was followed by the use of Western blot and cross-adsorption studies. An immunofluorescence assay was considered positive if immunoglobulin G (IgG) titers were >1:64 or if IgM titers were >1:32. When cross-reactions were noted between the rickettsial antigens, the analysis comprised 3 steps. First, a rickettsial antigen was considered to represent the agent of infection when IgG or IgM antibody titers against this antigen were >2 serial dilutions higher than titers of IgG or IgM antibody against other rickettsial antigens (*11*). Second, when the difference in titers between *R. felis* and other antigens was <2 dilutions, Western blot assays were performed. A rickettsial antigen was considered the agent of infection when sera reacted only against the specific protein of this antigen. Expected molecular masses of the specific proteins were ≈125 kDa for *R. typhi* and 31 kDa for *R. felis*. Finally, when Western blot assays were not diagnostic, cross-adsorption studies were performed, as previously described (*12*). Specific diagnosis criteria after cross-adsorptions studies included a Western blot assay that showed exclusive reactivity with specific proteins of a sole agent. If reactivity with the 2 tested agents was still observed, diagnosis of an indeterminate rickettsial disease was made. With this strategy, patients were classified by 3 types: *R. felis* infection, *R. typhi* infection, and indeterminate rickettsial disease.

Five fleas from the dog of 1 *R. felis*–infected patient were tested by polymerase chain reaction (PCR) (3). DNA was extracted and amplified with primers that targeted the citrate synthase sequence, as previously described (*3*). For negative controls, we used sterile water and infection-free fleas previously tested in our laboratory; both negative controls were tested after every 7 samples. Amplicons were separated by electrophoresis on 1% agarose gels and then purified by using a QIAquick PCR purification kit (Qiagen, Hilden, Germany), as described by the manufacturer. PCR products were sequenced by using the d-rhodamine terminator-cycle sequencing kit (PE Applied BioSystems, Courtabeuf, France), as described by the manufacturer. The sequences obtained were compared with those available in the GenBank DNA database by using the program Basic Local Alignment Search Tool (BLAST, version 2.0, National Center for Biotechnology Information (http://www.ncbi.nlm.nih.gov/BLAST/).

A rickettsial infection was diagnosed by using MIF for 31 of the 44 patients: 5 patients with the final diagnosis of *R. felis* infection, 13 with the diagnosis of *R. typhi* infection, and 13 with the diagnosis of indeterminate rickettsioses (Table). The diagnosis of *R. felis* infection was based on Western blot results on adsorbed sera for all patients. All the antibodies of these patients were removed when the *R. felis*–adsorbed sera were analyzed with *R. typhi* and *R. felis* antigens, whereas antibodies to *R. felis* remained when the *R. typhi*–adsorbed sera were analyzed.

**Table:** Clinical, epidemiologic, and biological data between Rickettsia felis group, R. typhi group, and indeterminate rickettsiosis group

Characteristic	*R. felis*	*R. typhi*	Indeterminate	p value
No.	5	13	13	–
Mean age, y*	45 (16)	29 (14)	40(17)	NS†
Sex (M/F)	5/0	10/3	12/1	NS‡
Contact with dogs or cats§	4/5	11/13	11/13	NS‡
Interval between clinical picture and evaluation, d¶	12 (9.5–14)	9 (8.5–10.2)	9 (7.9–13.4)	<0.05#
Fever§	5/5	13/13	13/13	NS‡
Maximal temperature (°C)*	39.3 (0.8)	39.6 (0.5)	39.4 (0.5)	NS†
Headache§	4/5	12/13	13/13	NS‡
Conjunctivitis§	1/5	3/13	2/13	NS‡
Arthralgia/myalgia§	4/5	6/13	5/13	NS‡
Odynophagia§	0/5	6/13	0/13	0.01‡
Dry cough§	3/5	8/13	2/13	0.04‡
Nausea/vomiting§	0/5	2/13	1/13	NS‡
Abdominal pain§	1/5	1/13	0/13	NS‡
Rash§	0/5	9/13	6/13	0.03‡
Past or actual tick bite§	1/5	2/13	1/13	NS‡
Hepatomegaly§	1/5	6/13	5/13	NS‡
Splenomegaly§	0/5	3/13	2/13	NS‡
Anemia (hemoglobin <13 mg/dL)	0/5	3/13	1/13	NS
Normal blood leukocyte counts (4,000–10,000/μL)	5/5	10/13**	11/13††	NS‡
Normal platelet counts (150,000–400,000/mL)	4/5	11/12‡‡	11/13‡‡	NS‡
Normal ratio prothrombin time (0.8–1.2)	4/4	10/13	11/13	NS‡
Normal ESR (<10 mm/h)	1/4	11/12	3/11	NS‡
Normal creatinine blood level (62–106 mmol/L)	5/5	11/13	10/13	NS‡
Normal sodium blood level (136–144 mmol/L)	2/4	10/13	10/12	NS‡
Elevated AST (>35 IU/L)	4/5	8/13	5/12	NS‡
Mean AST (U/L)	123	254	72	0.01§§
Elevated ALT (>45 IU/L)	5/5	8/13	6/12	NS†
Mean ALT (U/L)¶¶	185 (71–374)	354 (55–1,368)	86 (34–292)	<0.01§§
Elevated GGT (>55 IU/L)	2/5	3/13	4/12	NS‡
Elevated total serum protein concentration (>80 g/L)	0/5	0/12	1/12	NS‡
Elevated gamma globulin concentration (>13 g/L)	2/5	5/12	8/10	NS‡

*Data are expressed as mean (SD). Samples are distributed normally and have similar SD. NS, nonsignificant; ANOVA, analysis of variance test; ESR, erythrocyte sedimentation rate; AST, aspartate aminotransferase; ALT, alanine aminotransferase; GGT, gamma-glutamyl transpeptidase.

†ANOVA.

‡χ^2^ test.

§No. patients with these epidemiologic or clinical data/no. patients evaluated.

¶Data are expressed as median and 95% confidence intervals; >1 samples have a non-gaussian distribution.

#Significant differences between *R. felis* and *R. typhi* groups (p<0.05) with Dunn test.

**Leukopenia in 1 patient (3,700/μL), leukocytosis in 2 patients (11,500/μL and 16,000/μL).

††Leukopenia in 2 patients.

‡‡All patients with an abnormal platelet count presented with thrombocytopenia in all cases.

§§ANOVA; all cases.

¶¶Data are expressed as mean (range values). Samples are distributed normally and have similar SD.

Western blots performed with unadsorbed and adsorbed sera are represented in the Figure. Features of patients are indicated in the Appendix. Some differences were found between groups. The interval between the beginning of clinical signs and symptoms and evaluation was significantly more prolonged in the *R. felis* group than others. In the *R. typhi* group, odynophagia, cough, and rash were more frequent. When we compared biologic data, no difference was observed between *R. typhi* and *R. felis* groups, except for milder hypertransaminasemia in the latter group. Finally, 2 PCR products were obtained and sequenced from 2 fleas. Both sequences were 100% similar to *R. felis* citrate synthase gene in GenBank accession no. AF210692. No fleas were positive for *R. typhi*. Amplification was unsuccessful in all negative controls.

**Figure:**
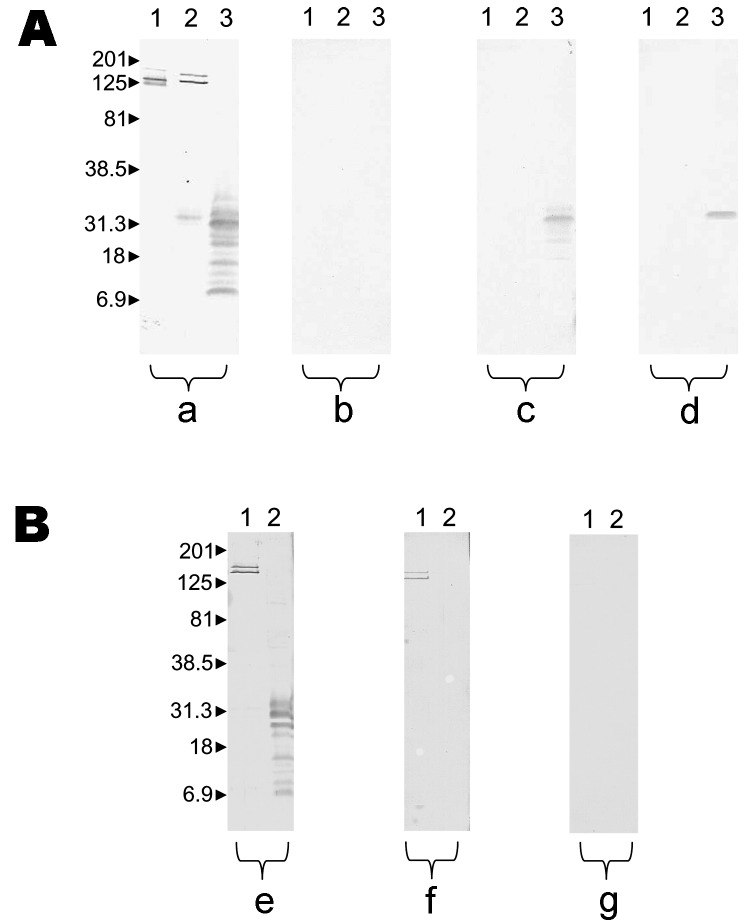
Results of Western blot performed with serum samples from patient 5 with Rickettsia felis infection and patient 10 with R. typhi infection. Molecular masses (in kilodaltons) are given to the left of panels. A) Patient with R. felis infection; a, untreated serum analyzed by using R. conorii (lane 1), R. typhi (lane 2), and R. felis (lane 3); b, R. felis–adsorbed serum analyzed by using R. conorii (lane 1), R. typhi (lane 2), R. felis (lane 3); all antibodies were removed; c, R. typhi–adsorbed serum analyzed by using R. typhi (lane 1) and R. felis (lane 2); antibodies to R. felis remained; d, R. conorii–adsorbed serum analyzed by using R. conorii (lane 1), R. typhi (lane 2), R. felis (lane 3); antibodies to R. felis remained. B) Patient with murine typhus; e, untreated serum analyzed by using R. typhi (lane 1) and R. felis (lane 2); f, R. felis–adsorbed serum analyzed by using R. typhi (lane 1) and R. felis (lane 2); antibodies to R. typhi remained; g, R. typhi–adsorbed serum analyzed by using R. typhi (lane 1) and R. felis (lane 2); all antibodies were removed.

## Conclusions

In the past 10 years, application of molecular tools has resulted in discovery of several new species of pathogenic rickettsiae, including *R. felis*. Since then, this bacterium was cultivated, and its genome was sequenced (*1**,**13*). Its pathogenic role was recently demonstrated in patients with serologic evidence of infection in Brazil, France, and Germany (*1*). *R. felis* DNA has also been detected in sera in Texas, Mexico, Brazil, and Germany (*1**,**4**,**8**,**14*). Autochthonous human rickettsioses that occur in the Canary Islands include murine typhus; SFG infections have never been reported (*9*). We diagnosed 5 cases of acute *R. felis* infection (*15*). The clinical picture is globally similar to murine typhus (*4*). However, the *R. felis* infection in our study seemed to be milder, and no skin rash was observed. The incidence of *R. felis* infection in the Canary Islands is probably underestimated; therefore, serologic tests for *R. felis* should be performed in patients with prolonged fever or suspected rickettsioses.

Cross-reactions in serologic testing for *R. felis* are unpredictable (*3*). In our study, patients with *R. felis* infection more frequently had high antibody titers (IgM >1:32 and IgG >1:64) to *R. conorii* and *R. typhi* (2 of 5 patients) than did patients with *R. typhi* infection (0 of 13). On the basis of *R. felis* data, we conclude that patients with *R. felis* infection may have no cross-reactivity with other rickettsiae, cross-reactivity with SFG rickettsiae, or cross-reactivity with both SFG rickettsiae and *R. typhi*. Genetic support for cross-reactivity with *R. conorii* is plausible because most membrane proteins of SFG and *R. felis* are extremely close (surface cell antigen [Sca] family). Genome analysis showed that several genes were present in *R. felis* and *R. typhi* and absent for other SFG, which could explain the cross-reactivity between *R. felis* and *R. typhi* (*13*). Finally, if <2-fold differences in IgG/IgM titers between *R. felis* and other SFG and typhus group rickettsiae are observed, only Western blot and cross-adsorptions will allow a specific diagnosis once reactivity has disappeared after adsorption with *R. felis* antigen. By contrast, a band of ≈31 kDa for the *R. felis* antigen persists after adsorption with *R. conorii* and *R. typhi*.
